# Review of Use Prevalence, Susceptibility, Advertisement Exposure, and Access to Electronic Nicotine Delivery Systems among Minorities and Low-Income Populations in the United States

**DOI:** 10.3390/ijerph192013585

**Published:** 2022-10-20

**Authors:** Susana Addo Ntim, Bria Martin, Yasmin Termeh-Zonoozi

**Affiliations:** Center for Tobacco Products, U.S. Food and Drug Administration, 11785 Beltsville Drive, Beltsville, MD 20705, USA

**Keywords:** environmental tobacco exposure from ENDS, race/ethnicity, socioeconomic status, tobacco-related inequities

## Abstract

Increased use of electronic nicotine delivery systems (ENDS) and improper disposal after use pose a public health and an environmental justice (EJ) concern if use prevalence is disproportionately high among minorities and people of low socioeconomic status (SES) (broadly termed “EJ populations” for the purposes of this review). This review synthesizes literature on demographic patterns of use prevalence, susceptibility, advertisement exposure, and access to ENDS, and extrapolates environmental tobacco exposure (ETE) from ENDS among EJ populations. Seven electronic databases were searched using ENDS-related terms. We included studies published between 2017 and May 2020 that described ENDS use prevalence, susceptibility to ENDS use, advertisement exposure, and access to ENDS by race, ethnicity, or SES. Data synthesis was based on the assumptions that ETE increases with high use prevalence, susceptibility may influence future use, and advertisement exposure and access may impact demographic differences in use. We identified 32 studies describing use prevalence, susceptibility, advertisement exposure, or access to vape shops and other tobacco retail outlets by race/ethnicity or SES. We found higher prevalence of ENDS use among non-Hispanic Whites and inconclusive use patterns by SES. Patterns of susceptibility to use, advertisement exposure, and access were also mixed, with slightly higher outcomes observed among low SES youth. However, the evidence base on advertisement exposure was limited, with limited generalizability. Our findings indicate low prevalence of ENDS use among EJ populations. While this suggests low potential ETE among these groups, mixed outcomes on susceptibility, advertisement exposure, and access to ENDS among low SES groups may affect future ENDS use and ETE. Educational campaigns that discourage ENDS uptake should target EJ youth. Initiatives aimed at managing vape shop presence in EJ communities and monitoring targeted advertisement are also needed.

## 1. Introduction

A sustained decrease in the prevalence of combustible tobacco smoking has been reported over recent decades [1]. However, disparities in tobacco use persist among racial/ethnic minorities and across groups defined by educational level and socioeconomic status (SES) [2]. Many studies have investigated the potential for electronic nicotine delivery systems (ENDS) to offer an alternative for those who struggle to quit, including smokers from socioeconomically disadvantaged backgrounds [3,4]. However, evidence from systematic reviews makes it difficult to draw firm conclusions about ENDS impact on smoking inequalities. Current ENDS use among United States (U.S.) adults declined from 3.7% in 2014 to 2.8% in 2017 and increased to 4.5% in 2019 [5,6]. A rapid increase in use among high/middle school students during 2017–2018 (11.7% to 20.8%/3.3% to 4.9%) prompted the Surgeon General to declare ENDS use among youth and young adults a public health epidemic [7,8]. The estimated 2021 current e-cigarette use among high and middle school students is 11.3% and 2.8%, respectively [9].

Increased use of ENDS presents the potential for environmental exposure to toxic chemicals from secondhand and thirdhand aerosols and ENDS waste, posing a public health concern. The presence of toxic chemical constituents in ENDS aerosols is well-documented. Ultrafine particles, nicotine, glycerin, and toxicants created by the oxidation or dehydration of vegetable glycerin and polyethylene glycol including formaldehyde and acetaldehyde are present in secondhand aerosols (SHA) from ENDS [10,11,12,13]. Recent literature suggests that SHA pollutant levels are generally lower than those in secondhand smoke (SHS). However, ENDS are still a source of indoor air pollutants because SHA toxicant levels are above background levels [13,14,15,16,17,18]. Much like thirdhand smoke, SHA can form residues on indoor surfaces; one study found SHA constituents from a vape shop traveled to form deposits on indoor surfaces in adjacent businesses [19].

The disposal of ENDS components may also pose a public health concern. Littered or improperly discarded ENDS can leach heavy metals, battery acids, and organic chemicals, potentially affecting humans and other organisms [20,21,22]. Small components (e.g., pods, cartridges) can pose choking hazards for small children and may be eaten by birds and other animals. Exploding ENDS batteries can cause severe burns and start fires.

These environmental tobacco exposure (ETE) scenarios may present an environmental justice (EJ) concern if ENDS use prevalence is disproportionately high among minorities and low-income populations (broadly termed “EJ populations” herein). EJ assessment was mandated under the National Environmental Policy Act (NEPA) by Executive Order (EO) 12,898 in 1994 to ensure equitable protection from environmental and human health hazards resulting from federal agency actions. The EO and associated Presidential Memorandum ordered the creation of the Interagency Working Group on Environmental Justice, which provides guidance to federal agencies on criteria to identify and address potential disproportionate human health, economic, and social effects, including effects on minority and low-income communities through the NEPA process [23].

Racial and ethnic disparities in ETE resulting from combustible tobacco product use are well-documented [24,25,26]. However, studies reporting sociodemographic differences in ETE from ENDS use are limited. This review synthesizes available scientific literature on use prevalence, susceptibility, advertisement exposure, and access to ENDS in the U.S. to address two questions: (1) Are use prevalence, susceptibility, advertisement exposure, and access to ENDS disproportionately high among EJ populations? (2) Are EJ populations likely to have disproportionately high environmental exposure to ENDS aerosols and wastes?

## 2. Methods

### 2.1. Search Strategy

The literature search covered seven databases of peer-reviewed articles using terms for all available ENDS data (Supplementary File S1). Initially, 8675 studies were identified between 2017 and May 2020. We screened titles and abstracts for articles focused on use prevalence, susceptibility, advertisement exposure, or access to ENDS, resulting in 264 abstracts.

### 2.2. Study Selection

**Figure 1:**
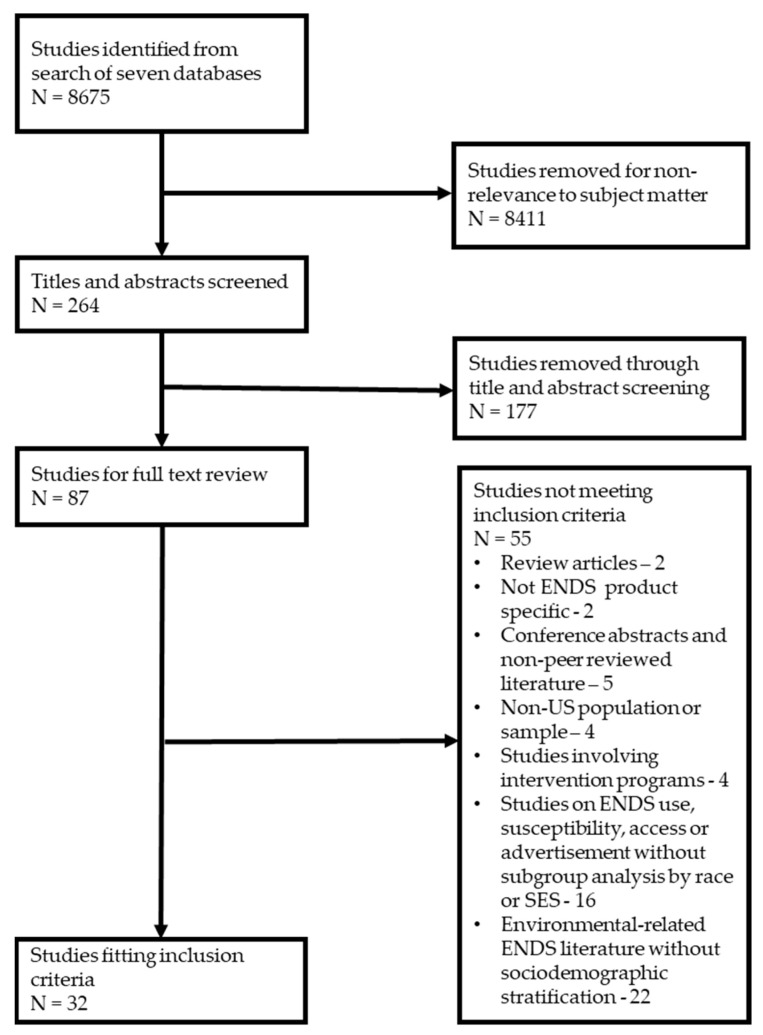
PRISMA flow diagram.

Studies were selected to answer the two questions noted above. We limited the review to publications from 2017 onwards, reflecting the period of a general increase in ENDS use [27]. No limits were set on the study design or scale, but we restricted our search to articles available in English. To meet our inclusion criteria, studies had to report use prevalence, susceptibility, advertisement exposure, or access to ENDS or a combination of those measures by race/ethnicity or an appropriate SES indicator (household income, user’s educational attainment, parent education, school setting) in a population or sample. We excluded non-peer-reviewed articles, studies involving intervention programs, review articles, and books. We also excluded non-U.S. studies to reflect NEPA’s reach. We assessed titles and abstracts against eligibility criteria after initial screening and removal of irrelevant references. A full-text review was conducted for 87 studies, including independent assessment by all authors and a secondary review of all exclusions. Discrepancies were resolved by discussion among authors. Our final evidence base comprised 32 studies (Figure 1).

### 2.3. Data Extraction

Data on the following factors were extracted: article and publication information, research questions, description of study population group with inclusion or exclusion criteria if applicable, demographics, SES indicators, study design, method summary, measured outcomes and results, study strengths and limitations including those listed by the authors, and risk of bias (Supplementary File S2).

### 2.4. Data Analysis

The review findings were summarized via narrative synthesis of selected studies. Racial/ethnic minorities were classified as all races other than non-Hispanic Whites (NHW). Meta-analyses were not performed due to study design diversity (longitudinal, cross-sectional, cross-sectional from longitudinal data, quantitative) and heterogeneity of study settings (national, state, county, local). We characterized potential exposure to SHA and chemicals from ENDS wastes as ETE. Direct estimation of ETE was not possible because studies directly describing ENDS-related ETE by race/ethnicity and SES are limited.

ETE was extrapolated from study findings based on three assumptions. First, potential ETE increases with use prevalence, and ENDS users are likely to use products in homes and vehicles around people of similar racial/ethnic and socioeconomic backgrounds. The literature supports this assumption: EJ populations have higher SHS exposure partly due to smoking rates and places of domicile and employment [28,29,30]. Second, ENDS use susceptibility may influence future use among non-users and experimental ENDS users. Longitudinal studies show that ENDS susceptibility among youth is a predictor of subsequent ENDS initiation and use [31,32,33,34]. Third, exposure to ENDS advertisements and access to vape shops and other tobacco retail outlets may impact demographic differences in ENDS use. Several factors are associated with future ENDS use, including product advertising, cigarette smoking, age, and being NHW [35]. Significant associations have also been reported between receptivity to tobacco advertising and progression toward use [34].

High ETE was identified when use prevalence, susceptibility, advertisement exposure, or access to ENDS was higher among at least one racial/ethnic minority group than among NHW, and among people of a low SES background. Low ETE was identified when use prevalence, susceptibility, advertisement exposure, or access to ENDS among racial/ethnic minority groups was less than or equal to exposure among NHW, and low among people of low SES backgrounds. Contradictory or complex use patterns, susceptibility, advertisement exposure, and access to ENDS were classified as unclear with respect to potential ETE.

## 3. Results

We identified 32 studies describing use prevalence, susceptibility, advertisement exposure, access to ENDS by race/ethnicity, or SES (Figure 1). Articles reviewed included studies analyzing cross-sectional and longitudinal data from national, state, and local surveys. All studies used self-reported outcome measures of unknown validity or reliability due to the lack of research to date on such measures. Various terms were used to describe the racial/ethnic makeup of study participants; for the purposes of this study, NHW represents all non-Hispanic Whites, non-Hispanic Black (NHB) represents Blacks and African Americans, and Hispanic represents Hispanics and Latinos. Other racial groups included Asians and Native Americans or Alaskan Natives. Key findings are summarized in Table 1.

Sixteen studies reported use prevalence among youth (12–17), young adults (18–25), and adults (18 and older). Fifteen of those studies reported subgroup analysis by race/ethnicity and ten by SES. Five studies reported these for “ever use” and seven for “current, past month, or past 30-day use.” Two studies reported ENDS use among current smokers. Definitions of “current use” and “ever use” were not consistent; “current use” was mostly defined as daily use or any use within the past 30 days or past month, while “ever use” was defined variously from any use in the past 12 months to ever lifetime use.

Nine studies evaluated ENDS susceptibility; seven reported on susceptibility by race/ethnicity and six by SES. In studies reporting subgroup analysis by race/ethnicity, one study reported results for ENDS and polysubstance use, one for ENDS and other tobacco products, and one for ENDS and cigarette use. One study evaluated harm perception as a factor in ENDS susceptibility by race/ethnicity and SES.

Thirteen studies evaluated advertisement exposure and ENDS access. Of these, four reported on both advertising and access, four reported advertising only, and five reported access only. For advertising, five studies reported subgroup analysis by race/ethnicity, and five studies by SES. For access, nine studies reported subgroup analysis by race/ethnicity and seven studies by SES.

### 3.1. Race/Ethnicity

#### 3.1.1. Prevalence of Use

**Figure 2:**
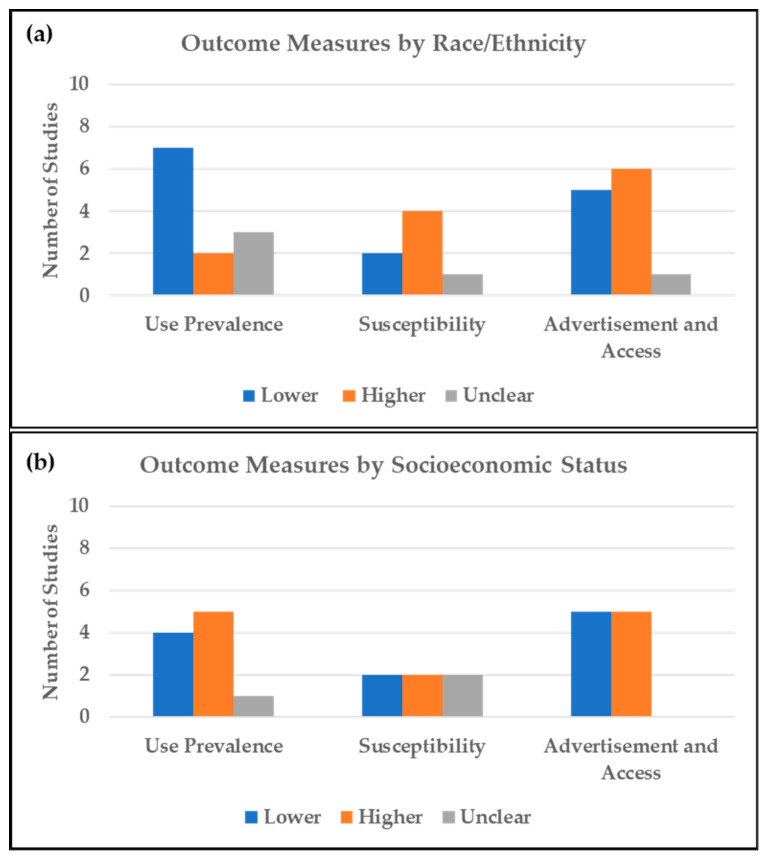
Distribution of studies showing the direction of potential environmental tobacco exposure (ETE) from ENDS among (**a**) racial/ethnic minorities compared to non-Hispanic whites, and (**b**) low socioeconomic status compared to high socioeconomic status. Higher: refers to higher potential for ETE among minorities and low SES groups. Lower: refers to lower potential for ETE among minorities and low SES groups. Unclear: contradictory or complex patterns of potential ETE.

Studies describing use prevalence by race/ethnicity generally showed lower prevalence among minorities compared to NHW (Figure 2a). Outcomes from surveys involving adult participants were similar to those involving youth with respect to use prevalence among racial/ethnic minorities. Seven studies reported lower use prevalence among racial/ethnic minorities [36,37,38,39,40]. Data from the 2014 and 2018 National Health Interview Survey (NHIS) showed an increase in ENDS ever use prevalence among U.S. adults for all races (13.0% to 15.7%), with the highest prevalence in 2018 observed among NHW (19.1%) [36]. Results from a national survey of high school students showed that schools with a higher percentage of NHW students (14.4% for tenth grade, 17.1% for twelfth grade) and schools with a higher prevalence of past-month cigarette smoking (adjusted odds ratio (AOR) = 6.82; 95% confidence interval (CI) = 5.68–7.96) had significantly higher past-month ENDS use [38]. Similarly, a nationally-representative youth survey found lower odds of ENDS poly-use (tobacco, alcohol, or cannabis) among all other racial/ethnic groups (odds ratios (ORs) = 0.18–0.61) compared to NHW [37]. Other studies reporting lower ENDS use among minorities involved data from local and regional surveys [39,40]. A study involving Los Angeles County adults reported the highest prevalence of ENDS ever use among NHW adults (12.8%) followed by Asian adults (8.9%); NHB had the lowest prevalence (5.8%) [39]. A study assessing JUUL use among ever and past-30-day ENDS users reported a positive association between JUUL use and being NHW [40]. Four studies reported higher use prevalence among minorities; one of these analyzed data from a national survey [41,42,43]. Three of those studies reported higher odds of ENDS ever use among Hispanic adolescents compared to NHW (3% vs. 1.5%, *p* = 0.003; 34.5% vs. 24.9%, *p* < 0.001; and OR = 1.30, 95%CI = 1.17–1.45) [41,42,43] and one reported the highest ENDS use among non-Hispanic “other” adults (OR = 9.3, 95%CI = 6.0–12.6) compared to non-Hispanic Whites [6]. However, lower odds of ENDS ever use were reported among NHB (OR = 0.72, 95%CI = 0.63–0.83) and Asian (OR = 0.64, 95%CI = 0.50–0.81) students compared to NHW [41]. Results from three nationally representative studies were mixed [5,44,45]. JUUL ever use prevalence among participants aged 15–34 was comparable between Hispanic and NHW ENDS ever users (14.6% vs. 14.4%) [44]. Similarly, self-reported current and regular e-cigarette use were the highest among non-Hispanic whites and persons of “other” races [5,45].

#### 3.1.2. Susceptibility to ENDS Use

Studies examining the susceptibility by race/ethnicity generally reported higher susceptibility among minority youth and lower susceptibility among minority adults (Figure 2a). Four studies reported higher susceptibility among racial/ethnic minorities. Results from a study analyzing a nationally representative sample of participants aged 13–18 revealed that perceived health risks of nicotine and toxins in ENDS products were 34% lower in NHB compared to NHW [46]. A study following use patterns in adolescents reported that baseline ENDS ever use was higher for Hispanics than NHW (34.5% vs. 24.9%; *p* < 0.001) but lower at a 12-month follow-up for past 30-day use (17.3% vs. 13.2%; *p* < 0.001) [42]. Susceptibility was significantly higher among Hispanic compared to NHW youth in two studies in Nevada (AOR = 1.89; 95%CI = 1.27–2.83) and in central Texas (38.7% vs. 29.7%; *p* < 0.0001) [43,47]. Two studies reported lower susceptibility among minorities; both involved national surveys (OR (NHB) = 0.27, 95%CI = 0.09–0.77; OR (Hispanic) = 0.26, 95%CI = 0.09–0.70) [37,48]. Evidence from one study indicated mixed or unclear outcomes with respect to susceptibility. The results revealed that, among current users of noncigarette combustible tobacco, Hispanics and NHB adults were more likely to use ENDS (OR (NHB) = 2.7, 95%CI = 1.0–4.3; OR (Hispanic) = 3.3, 95%CI = 1.2–5.4). However, among current cigarette smokers, racial groups other than NHB were more likely to use ENDS, indicating that susceptibility among racial groups may differ by combustible product use status [49].

#### 3.1.3. Advertisement Exposure and Access to ENDS

Outcomes from studies describing advertisement exposure and access to ENDS by race/ethnicity were mixed (Figure 2a). Five studies reported on advertising or marketing strategies by race/ethnicity [48,50,51,52,53]; two analyzed data from national surveys [48,51]; and three involved data from county and state surveys [50,52,53]. Nine studies reported ENDS access by race/ethnicity [50,52,53,54,55,56,57,58,59]; six analyzed state and local data [50,52,53,54,55,59]; and three analyzed nationally representative data [56,57,58].

For advertising, a study analyzing data from Los Angeles County reported that marketing strategies differed by race/ethnicity, and exterior advertising was less prominent in Hispanic/Latino (OR = 0.36, 95%CI = 0.17–0.72) and Korean American (OR = 0.28, 95%CI = 0.10–0.74) communities compared to NHW communities [50]. ENDS advertising was significantly lower in neighborhoods with majority NHB (20.3%, adjusted prevalence ratio (aPR) = 0.63(0.41,0.99)) and Hispanic (22.9%, aPR = 0.62(0.40,0.98)) populations in New York City (NYC) [53]. E-cigarette advertising declined in retail stores close to high schools in NJ including districts with >50% non-White students, between 2015 and 2016 [52]. In two national studies, NHB youth reported higher ENDS use following higher exposure to advertising or marketing strategies at rates over 2.5 and 3 times higher than Hispanic and NHW youth, respectively [51]. Among adults, NHB (30.10%, *p* < 0.05) and “other” (non-Hispanic other) (17.42%, *p* < 0.05) users reported higher use due to appealing advertising [48].

Studies on access were mostly at state and county levels and revealed mixed results. ENDS were less likely to be placed in proximity to youth-friendly items in African American (OR = 0.32, 95%CI = 0.16–0.65), Korean American (OR = 0.20, 95%CI = 0.07–0.59), and Hispanic/Latino (OR = 0.07, 95%CI = 0.02–0.26) communities in Southern California, and retailers were less likely to sell and advertise ENDS, compared to NHW communities [50]. In NJ, e-cigarette availability near high schools, including school districts with >50% non-White students, decreased over a one-year period (2015–2016) [52]. Austin, Texas had lower proportions of NHB (5.7%) and Hispanic (32.7%) residents in census tracts with a vape shop compared to NHW residents (77.7%). Though the odds of finding a vape shop in Hispanic areas was high, the odds were low for NHB areas (AOR = 0.90; 95%CI = 0.815–0.997) and decreased with an increasing percentage of NHB residents [54]. However, results from a study in Orange County, California revealed that census tracts with at least one vape shop had a higher percentage of Asians (mean = 20.1%, *p* = 0.030), Hispanics (mean = 35.8%, *p* = 0.001), and people born outside the U.S. (mean = 31.8%, *p* = 0.004) [55]. Tobacco retailers in NYC had the lowest ENDS availability in high percent NHB (28.7%, aPR = 0.71(0.51,0.98)) and Hispanic (28.3%, aPR = 0.75(0.53,1.05)) neighborhoods [53]. Vape shops were more likely to be located in neighborhoods with high Hispanic populations across Virginia [59]. Similarly, national studies on vape shop proximity to public middle and high schools revealed that vape shop density was higher and vape shops were closer to schools in districts with higher proportions of Asian and NHB populations [56]. Another national study found vape shops were more likely to be in urban areas with high Hispanic (adjusted risk ratio (aRR) = 3.3, *p* < 0.0001) and Asian (aRR = 2.0, *p* < 0.0001) populations, and non-urban areas with high African American (aRR = 3.9, *p* = 0.0009) and Hispanic (aRR = 7.4, *p* < 0.0001) populations [58]. Xiao et al. suggested that increasing ENDS prices may decrease demand more among other non-Hispanic ethnicities compared to NHWs due to potential affordability-driven use (AOR = 2.684, 95%CI = 1.044–6.899) [57]. However, non-Hispanic “other” (89.30%), NHW (78.79%), and Hispanic (76.59%) youth reported higher ENDS use due to appealing flavors than NHB youth (53.62%) [57].

### 3.2. Socioeconomic Status

#### 3.2.1. Prevalence of Use

Outcomes from studies describing ENDS use prevalence by SES were mixed (Figure 2b) [39,40,44,60,61,62]. Four studies reported lower use prevalence among low SES groups [39,40,44], five studies reported higher prevalence among low SES groups [60,61,62], and one study reported unclear results [45]. Among the studies reporting low prevalence among low SES groups, one study reported higher ENDS ever use among adults with a household income ≥300% of the federal poverty level (FPL) compared to those = 0–99% FPL (10.7% vs. 6.3%), and among adults with some college education or higher compared to those with less than high school education (10.2% vs. 4.6%) [39]. Self-reported current e-cigarette use significantly increased from 2017 to 2018 among U.S. young adults and adults with income four times the FPL or greater (difference = 4.3%, 95%CI = 0.6–8.0%, *p* = 0.008) [5]. JUUL use was reportedly highest among ENDS ever users with high SES backgrounds and among people living in the northeast (17.1% ever users, 7.8% current users) [40,44]. Among studies reporting high prevalence among low SES groups, a national survey showed higher odds of use among individuals with less than high school education (OR = 1.47, 95%CI = 1.08–2.00) and among people with income below FPL (OR = 1.31, 95%CI = 1.01–1.69) [60]. Results from a 2019 national survey showed the highest ENDS use among those in the lowest income bracket (OR = 5.0, 95%CI = 4.4–5.6) and those with no more than a high school education (OR = 7.8, 95%CI = 5.5–10.1) [6]. Higher education was linked with lower odds of using ENDS, with the significant inverse association observed among NHW but not NHB adults (OR = 0.76, 95%CI = 0.61–0.95) [61]. Compared to non-users in a 2013–2014 national survey, e-cigarette users were more likely to be less educated or have a lower income [63]. A study describing poly-product use among adolescents showed that lower parental education was associated with increased odds of past ENDS use (OR = 1.30, 95%CI = 1.12–1.51), and lower school subjective social status was associated with increased odds of past (OR = 1.11, 95%CI = 1.03–1.20) or current use (OR = 1.25, 95%CI = 1.08–1.44) of ENDS compared to never use [62]. Results from a 2014 national survey indicated that regular e-cigarette use varied by educational level but had no significant association with income [45].

#### 3.2.2. Susceptibility to ENDS Use

Studies examining ENDS susceptibility by SES are inconclusive (Figure 2b). Two studies reported higher susceptibility among low SES youth, two reported lower susceptibility among low SES adults, and two reported unclear results [43,46,48,49,61,64]. Studies reporting higher susceptibility among low SES youth revealed lower health risk perceptions of nicotine and ENDS toxins or chemicals among youth with lower parental education (OR = 0.69, 95%CI = 0.55–0.87) and low-income status (OR = 0.72, 95%CI = 0.58–0.91). Additionally, students in lower SES schools had significantly higher odds of susceptibility compared with students at higher SES schools (AOR = 2.01, 95%CI = 1.49–2.71) [43,46]. Studies reporting lower susceptibility among low SES participants revealed that lower income smokers were more likely to believe ENDS are more harmful than cigarettes (OR = 1.40, 95%CI = 1.08–1.82) [48]. Lower SES was associated with a reduced overall likelihood of ENDS use among adults (OR = 1.01, 95%CI = 0.83–1.24) and higher education was associated with a 0.9% increase in the likelihood of switching from conventional cigarette smoking to ENDS compared to those without any higher education (95%CI = 0.0–1.9) among ever smokers [48,64] Evidence from one study analyzing nationally representative data revealed that among current cigarette smokers, those living at or above the FPL with higher education were more likely to use ENDS (OR = 60.7, 95%CI = 57.9–65.3, OR = 61.7, 95%CI = 58.4–64.9). However, results also revealed that among current users of noncigarette combustible tobacco, those living in poverty were more likely to have ever used ENDS (OR = 4.1, 95%CI = 1.5–6.8) [49]. Another study revealed an inverse association between education attainment and ENDS use in NHW but not in NHB adults (OR = 1.63, 95%CI = 1.04–2.56) [61].

#### 3.2.3. Advertisement Exposure and Access to ENDS

Studies examining ENDS advertisement exposure and access by SES generally reported higher exposure and lower access among low SES youth (Figure 2b). Three studies reported on ENDS advertising and marketing strategies by SES [51,57,65], and three described access by SES [54,55,56].

For ENDS advertising, a survey of Connecticut high school students reported higher potential for high SES youth to experience greater recent advertising exposure, indirectly influencing e-cigarette use (indirect effect: β = 0.01, standard error (SE) = 0.00, 95%CI = 0.001–0.010, *p* = 0.02) [65]. However, a study analyzing nationally representative data revealed that low SES youth were also more likely to report using ENDS because people in the media or other public figures used them than those in higher SES groups (52.3%, 95%CI = 40.21–64.13) [51]. Similarly, a later study reported that those with household income <$50,000 were more likely to use ENDS because the advertising appeals to them (~58%) [57].

For access to ENDS, three studies revealed that increased poverty correlated with increased odds of vape shop presence in Austin, TX (AOR = 1.07, 95%CI = 1.010–1.125), Orange County, CA (%poverty in areas with at least one vape shop (12.4%) vs. areas with none (8.8%), *p* < 0.001), and in neighborhoods across Virginia with low household income, a higher percentage of renter-occupied housing, lower gross rent cost, and a higher percentage of vacant houses [54,55]. However, studies assessing the nationwide distribution of vape shops revealed that shops were further away from schools in school districts in higher-poverty areas [56], or that poverty was not a significant determinant of vape shop density [58]. In NYC and NJ, ENDS availability was highest in high-income neighborhoods (64.3%, aPR = 1.00 (reference)) [53], and in retail stores close to mid-to-high income school districts, respectively [52].

## 4. Discussion

Studies directly describing environmental exposure to secondhand and thirdhand ENDS aerosols and chemicals from ENDS waste on EJ populations are limited. This narrative review attempts to extrapolate demographic differences in potential ETE from use prevalence, susceptibility, advertising exposure, and access to ENDS. We synthesized available studies to assess use prevalence among minorities compared to NHW, and people of low vs. high SES backgrounds. Susceptibility to ENDS use, advertisement exposure, and access was also reviewed, since susceptibility may influence future use among non-users and experimental users, and advertising and access may influence demographic differences in ENDS use. The potential for ETE was extrapolated from reviewed data based on those assumptions.

Generally, our review showed lower potential ETE among racial/ethnic minorities compared to NHW with respect to ENDS use prevalence. This is consistent with findings from studies showing a higher likelihood of exposure to SHS and SHA among NHW youth [66,67,68]. Similarly, a recent review on sociodemographic differences in ENDS awareness and use showed that ENDS appear to have gained greater reach among NHW than other racial/ethnic groups, and among people with higher educational attainment [69]. However, our review revealed inconclusive results on use prevalence and SES. Lucherin et al. reviewed the potential for non-combustible nicotine products to reduce socioeconomic inequities from smoking and reported a positive association between ENDS use prevalence and high SES; they acknowledged that the evidence suggests a potential flattening of the SES gradient over time [2]. The mixed outcomes revealed in our review may also be indicative of a potential leveling of the SES gradient. Lower use prevalence among minorities presents a positive outlook on potential ETE from ENDS and does not show disproportionately high ETE among those groups. However, mixed outcomes observed with respect to use prevalence by SES and the potential flattening of the SES gradient may affect the future ETE outlook among these groups.

We found mixed results from studies assessing ENDS use susceptibility. While these studies were more likely to suggest higher susceptibility among low SES and minority youth, the opposite was reported for low SES and minority adults. High susceptibility among minority youth is concerning, because ENDS susceptibility is a potential predictor of future ENDS use and use of products such as marijuana and alcohol [70]. Though ENDS susceptibility trends cannot be compared to ENDS use trends with cross-sectional data, longitudinal studies have found that ENDS susceptibility predicts subsequent use at follow-up [7,31,32,34]. Longitudinal data would clearly characterize the influence of susceptibility on ENDS use and associated demographic factors.

Advertising exposure and access were higher among racial/ethnic minority youth and adults. However, differential advertisement exposure and access to ENDS were mostly reported at state and local levels, though these data may lack generalizability. Advertisement exposure and access also appeared to be influenced by SES. However, the current evidence on sociodemographic differences in advertisement exposure and access was limited; additional studies can help better understand ENDS use resulting from advertisement exposure and access, and associated ETE among EJ groups. Initiatives aimed at controlling advertising may help curb youth e-cigarette use [35]. FDA’s youth tobacco prevention campaigns [71] and the surgeon general’s call for aggressive state and local actions (e.g., restricting youth access to ENDS in retail stores, including ENDS in smoke-free indoor air policies) [8] are important steps to reduce initiation, use, and ETE potential. FDA’s enforcement policy on flavored cartridge-based ENDS [72] and flavored ENDS bans by state and local governments [73] may also curb future initiation.

## 5. Limitations

To our knowledge, our review is the first to infer sociodemographic patterns of potential ETE from ENDS use, susceptibility, advertising, and access. However, several limitations exist. We did not assess study quality. As studies directly examining ENDS-related ETE are limited, we relied on several assumptions to extrapolate ETE from the studies reviewed. The direct assessment of ETE through surveys and measurement of biomarkers of exposure could provide more reliable data to inform EJ assessments through the NEPA process. As with all reviews, we were limited by the evidence available and its reporting. For instance, most studies involved self-reported surveys; therefore, recall bias and other individual study limitations may have impacted our results. Heterogeneity in study design and settings made meta-analysis impossible. Some studies included local and regional surveys; sociodemographic characteristics differ widely across regions and observations in any one region may not be generalizable. Furthermore, differences in exposure and use pattern measurement made it difficult to draw conclusions. For example, some studies assessed ENDS use only while others assessed dual, or poly-use across subgroups. Many studies did not consider differences in ENDS product preferences by brand, type, flavor, nicotine concentration, cost, or other characteristics, potentially skewing subgroup results. Finally, the ENDS landscape is quickly changing; the sociodemographic patterns reported may have evolved beyond our search time frame.

## 6. Conclusions

Our review extrapolates the potential for disproportionately high ETE among EJ populations resulting from ENDS use prevalence, susceptibility, advertisement exposure, and access. The findings indicate that ENDS use is less prevalent among EJ populations. While this suggests a low potential for ETE among these groups, mixed outcomes on susceptibility, advertisement exposure, and access among low SES groups show a potential flattening of the SES gradient over time, which may affect future ENDS ETE. These findings underscore the importance of educational campaigns to prevent initiation and subsequent addiction among non-users of tobacco products and initiatives aimed at managing vape shop presence in EJ communities and monitoring targeted advertisements.

## Figures and Tables

**Table 1:** Key findings from the articles.

Citation	Data Source	Study Setting	Sample Size & Age Group	Study Design	Outcome Measure	Findings
Assari et al., 2020	2017 HINTS	National	2277 adults aged ≥ 18	Cross-sectional	Use prevalence; susceptibility	Overall, a higher level of educational attainment is linked to lower odds of ENDS use (OR = 0.76, 95%CI = 0.61–0.95). By race, inverse association between education attainment and ENDS use in NHW adults (OR = 1.63, 95%CI = 1.04–2.56). No significant interaction between educational attainment and ENDS use for NHB individuals.
Barrington-Trimis et al., 2019	Spring 2014 CHS, Fall 2015 H&H, and Fall 2013 YASS	Regional	Baseline: CHS-1553; H&H-3190; YASS-1404	Prospective cohort	Use prevalence; susceptibility	Baseline ENDS ever use higher for Hispanics (34.5%) than NHW (24.9%; *p* < 0.001). At follow-up, non-Hispanic White participants were more likely to report past 30-day use of any tobacco product relative to Hispanic Whites (17.3% vs. 13.2%; *p* < 0.001). Higher odds of stable tobacco use patterns observed among Hispanics. Unlike non-Hispanic White, Hispanic exclusive ENDS users had no increased odds of exclusive cigarette use.
Bello et al., 2019	Survey of 10 public high schools in greater Los Angeles, CA	Local	4100 students not enrolled in English as a second language programs or special education courses	Cross-sectional data from a longitudinal study	Use prevalence	Current and lifetime poly nicotine product use inversely associated with parental education and school subjective social status (SSS) (ORs:0.80 (95%CI:0.72–0.88) to 1.71 (95%CI:1.24–2.35)). Parental education was inversely associated with increased odds of past ENDS use (ORs: 1.30 (95%CI:1.12–1.51) to 1.46 (95%CI:1.16–1.83)). Lower school SSS associated with increased odds of past or current use of cigars, ENDS, prescription stimulants, and prescription pain killers (ORs:1.11 (95% CI: 1.03–1.20) to 2.03 (95% CI:1.57–2.62)). No significant associations between societal SSS and specific product use. All odds are relative to never use.
Bostean et al., 2018	2010–2014 Census tract, 2016 CA BOE, systematic internet search (2015)	County	Vape shops—163; census tracts—572; median population density—7400 persons per square mile	Geographical information and statistical analyses	Access	Higher % Asians (median of 15.5% vs. 12.7%, *p* < 0.05) and significantly higher median % Hispanics (33.7% vs. 19.3%, *p* = 0.001) in tracts with vape shops compared to tracts with none. Higher percent population of Asians (mean = 20.1%, *p* = 0.030), Hispanics (mean = 35.8%, *p* = 0.001), and people who were born outside the U.S. (mean = 31.8%, *p* = 0.004) with at least one vape shop. Significantly higher median % foreign-born (31.6% vs. 25.0%, *p* < 0.001), higher % poverty (12.4% vs. 8.8%, *p* < 0.001), and lower % college educated and above (20.4% vs. 25.9%, *p* < 0.001) in tracts with at least one vape shop compared to tracts with none. Higher population density associated with lower vape shop count. Significant association between vape shop count and % Hispanic, intermediate poverty, after adjusting for sociodemographic factors.
Chido-Amajuoyi et al., 2020	Google maps, Yelp, Yellow pages; 2014 Census Tract; TEA	Local	Vape shops-52. Census tracts-200. Population estimates—811,456 (2010) and 947,890 (2016)	Spatial and statistical analyses	Access	20% of tracts had at least one vape shop, seven census tracts had more than one vape shop. 37.5% of the tracts with vape shop met the criteria for classification as poverty areas, that number was 26.3% for vape shop free census tracts. NHW predominated areas with (77.7%) or without (75.1%) vape shops. Lower proportion of NHB (5.7%) and Hispanics (32.7%) in tracts with at least one vape shop compared to NHW residents (77.7%). 88% of vape shops were within one-mile radius of middle or high schools. Poverty was positively associated with vape shop presence (adjusted odds ratio (AOR), 1.07; 95%CI, 1.010–1.125). Percent population of NHB was inversely associated with vape shop presence (AOR, 0.90; 95%CI, 0.815–0.997).
Cornelius et al., 2019	2019 NHIS	National	31,997 adults aged ≥ 18	Cross-sectional	Access	Reported e-cigarette use prevalence highest among those in the lowest income bracket (OR = 5.0, 95%CI = 4.4–5.6), those with no health insurance (OR = 7.2, 95%CI = 6.1–8.3), those with no more than a high school education (OR = 7.8, 95%CI = 5.5–10.1), and those who identified as non-Hispanic “other” (OR = 9.3, 95%CI = 6.0–12.6).
Dai et al., 2017	Yelp.com, Yellowpages.com, Guidetovaping.com; Census Data—2014 ACS	National	Vape shops—9943; census tracts—72,758	Geographical information and statistical analyses	Access	Overall—vape shops more likely in urban than non-urban census tracts (mean density: 0.47 vs. 0.23; *p* > 0.05). Urban areas—larger proportions of Hispanics (adjusted risk ratio (aRR) = 3.3, *p* < 0.0001), Asians (aRR = 2.0, *p* < 0.0001), young adults 18–29 (aRR = 1.8, *p* = 0.0002)), and adults 30–44 (aRR = 9.0, *p* < 0.0001)) in tracts with higher vape shop density; lower vape shop density associated with youth under 18 (aRR = 0.5, *p* = 0.2) and tracts with higher education (≥college: aRR = 0.5, *p* < 0.0001). Non-urban areas—higher proportions of African Americans (aRR = 3.9, *p* = 0.0009) and Hispanics (aRR = 7.4, *p* < 0.0001) in tracts with higher vape shop density. Lower vape shop density in tracts with larger household size and higher percent owner-occupied housing in both urban and nonurban areas. No statistically significant association between vape shop density and percent poverty in both urban and non-urban areas.
Dai and Leventhal 2019	2014–2018 NHIS	National	2014 (*n* = 36 697), 2015 (*n* = 33 672), 2016 (*n* = 33 028), 2017 (*n* = 26 742), 2018 (*n* = 25 417). Adults aged 18–24, 25–44, 45–64, and ≥65	Cross-sectional	Use prevalence	Self-reported e-cigarette current use prevalence: Overall—2014 = 3.7%, 2015 = 3.5%, 2016 = 3.2%, 2017 = 2.8%, 2018 = 3.2%. Young adult—2014 = 5.1%, 2015 = 5.2%, 2016 = 4.7%, 2017 = 5.2%, 2018 = 7.6%). Overall, changes in current and daily e-cigarette use differed by age. Significant quadratic trend for prevalence of reported current e-cigarette use over 2014–2018, overall (*p* = 0.03), and among young adults. Significant 2017–2018 biannual increase in reported current e-cigarette use among young adults (difference = 2.4%, 95%CI = 0.4–4.4%), and among young adult who were former smokers (difference = 20.0%, 95%CI = 6.7–34.9%, *p* = 0.01), men (difference = 3.8%, 95%CI = 0.7–7.0%, *p* = 0.02), non-Hispanic whites (difference = 3.5%, 95%CI = 0.9–6.2%, *p* = 0.001), persons of other races (difference = 5.5%, 95%CI = 0.5–10.4%, *p* = 0.02), and those with poverty ratio of 4.0 or greater (difference = 4.3%, 95%CI = 0.6–8.0%, *p* = 0.008).
Du et al., 2019	2015 LACHS	County	7919 adults aged ≥ 18	Cross-sectional	Use prevalence	Age adjusted prevalence of ever ENDS use: overall—8.4%, higher among males; highest among NHW (12.8%) followed by Asians (8.9%), lowest among blacks (5.8%); highest among some college education or higher (10.2%); highest among household income ≥300% FPL (10.7%; however, no significant association reported AOR (95%CI) = 1.05 (0.73, 1.51)). Significantly high associations between ENDS ever use and some college education or higher, marijuana use, alcohol drinking, and current or former cigarette smoking (AOR (95%CI) = 1.52 (1.02, 2.26); 1.73 (1.31, 2.28); 1.80 (1.33, 2.42); 9.40 (6.94, 12.75), respectively). Blacks or foreign-born participants were significantly less likely to have ever used ENDS (AOR (95%CI) = 0.47 (0.28, 0.79); 0.56 (0.42, 0.75), respectively).
Escobedo et al., 2019	LA county census tract; CDTFA list	County	775 retail stores across AA (*n* = 194), HL (*n* = 189), NHW (*n* = 196), KA (*n* = 100) and AI (*n* = 96) communities	Geographical information and statistical analyses	Advertisement	Stores across all communities less likely than NHW communities to sell ENDS and flavored ENDS (OR (AA) = 0.24, 95%CI = 0.15–0.37, OR (KA) = 0.19, 95%CI = 0.11–0.33, OR (HL) = 0.09, 95%CI = 0.06–0.15), and to have self-service ENDS displays. Compared to NHW communities, exterior advertising less prominent in HL (OR = 0.36, 95%CI = 0.17–0.72) and KA (OR = 0.28, 95%CI = 0.10–0.74) communities. ENDS proximity to youth friendly items less likely in AA (OR = 0.32, 95% CI = 0.16–0.65), KA (OR = 0.20, 95%CI = 0.07–0.59), and HL (OR = 0.07, 95% CI = 0.02–0.26) communities than NHW. Significant difference in ENDS pricing (cheapest ENDS cost significantly less in AA than in KA communities). No other comparison was significant.
Friedman and Horn 2019	2014–2016 NHIS	National	50,306 adults aged 25–54	Cross-sectional	Susceptibility	Education and income gradients are flat for dual use (conventional cigarettes and ENDS) (−1.4 (CI = 1.8−0.9) and −1.9 (CI = 2.5−1.2)) and statistically insignificant for exclusive ENDS use ((−0.03 (CI = 0.5, 0.4) and −0.3 (CI = 0.8−0.2)). Negative education and income gradients for conventional cigarette use (CI = 14.0−11.8) if college educated and −9.5 percentage points (CI = 10.9−8.1) if household income exceeds 400% of the FPL.
Gilbert et al., 2020	2017 YRBSS	National	11,244 high school students (9th–12th grade) in public and private schools	Prospective and cross-sectional	Use prevalence; susceptibility	Lower odds of ENDS poly-use (ENDS use combined with another tobacco, alcohol, or cannabis) among racial/ethnic minorities compared to NHW youth (ORs = 0.18–0.61). Bisexual youth more likely to be ENDS poly-users compared to heterosexual youth (OR = 1.62). ENDS poly-users increased from 9th grade (7.1%) to 12th grade (16.3%). Significant positive relationship between poly-use status and frequency of ENDS use (F = 4.32, *p* = 0.01).
Giovenco et al., 2018	2014 NJ YTS	State	194 tobacco retailers within 0.5-mile radius of 41 high schools in NJ (a representative probability sample of NJ youth)	Geographical information and statistical analyses	Access; advertisement	E-cigarette availability declined across all store types and school districts, except chain convenience stores and drugs store, where no changes were observed between 2015 and 2016. Cigar/cigarillo availability increased across all store types and school districts, except in chain convenience stores, drug stores, and school districts with <50% non-White students. Accessibility and promotion of e-cigarettes and smokeless tobacco was more common in mid-to-high-income districts and schools with <50% non-White students. E-cigarette exterior advertising declined across all school districts and store types except drug stores; interior advertising declined across all school districts and store types, except chain convenience stores.
Giovenco et al., 2019	Census tracts, 2015 American Community Survey	Local	796 tobacco retailers in New York City	Geographical information and statistical analyses	Access; advertisement	Neighborhoods with the highest percentage of NHB residents had the lowest ENDS availability in tobacco retailers (28.7%, aPR = 0.71 (0.51, 0.98)). ENDS advertising was significantly lower in neighborhoods where NHB (20.3%, aPR = 0.63 (0.41, 0.99)) and Hispanic (22.9%, aPR = 0.62 (0.40, 0.98)) residents were the racial/ethnic majority. For NHB (68.2%, aPR = 1.59 (1.19, 2.11)) and Hispanic (66.8%, aPR = 1.54 (1.14, 2.08)) majority neighborhoods, tobacco retailers were significantly more likely to sell 99-cent cigarillos. By median household income, the highest quartile ($75,006–$170,766) had the highest availability (64.3%, aPR = 1.00 (ref)) of ENDS products at tobacco retailers.
Harlow et al., 2019	2016–2018 PATH W2	National	7219 adults aged ≥ 18	Longitudinal	Susceptibility; advertisement	NHB (OR = 0.27, 95%CI = 0.09–0.77) and Hispanic (OR = 0.26, 95%CI = 0.09–0.70) adults were less likely than NHW adults to become exclusive ENDS users. Lower income cigarette smokers (<100% FPL) were less likely to use ENDS (OR = 1.01, 95%CI = 0.83–1.24) and more likely to believe ENDS are more harmful than cigarettes (OR = 1.40, 95%CI = 1.08–1.82). NHB (30.10%, *p* < 0.05) and non-Hispanic “other” (17.42%, *p* < 0.05) ENDS users were more likely to use ENDS due to appealing advertising. Lower SES was associated with reduced overall likelihoods of ENDS use among adults (<100% FPL:OR = 1.01, 95%CI = 0.83–1.24).
Jaber et al., 2018	2013–2014 NHANES	National	10,175 participants (125 current e-cigarette users), 5423 adults aged ≥ 18 (116 e-cigarette users); 895 adolescents aged 13–17 (9 e-cigarette users)	Cross-sectional	Use prevalence	Self-reported e-cigarette and other tobacco products use were modified by smoking status and differed among demographic characteristics. Self-reported past 5 days e-cigarette use prevalence: weighted overall adult—2.6%, 95%CI = 2.0–3.1, adolescent (13–17)–1.21%, 95%CI = 0.3–2.1. Recent e-cigarette use prevalence highest among current smokers (8.2%, 95%CI = 6.3–10.1), followed by former smokers (2.7%; 95%CI = 1.4–4.1), lowest among never smokers (0.4%; 95%CI = 0.2–0.6). Lowest e-cigarette use prevalence among NHB (1.5%, 95%CI = 0.5–2.4), followed by Mexican Americans (1.6%, 95%CI = 0.1–3.1). E-cigarette users had lower odds of having a household income ≥ $75,000 (OR = 0.23, 95%CI = 0.7–0.79, *p* = 0.02) or having a college education (OR = 0.28, 95%CI = 0.15–0.54, *p* < 0.01) compared with never users of tobacco.
Levy et al., 2017	May 2014, Tobacco Use Supplement Survey—Current Population Survey	National	158,626 adults (aged 18–65+ years)	Ecological (analyzing cross-sectional data)	Use prevalence	Regular e-cigarette use highest among White (reference variable) and “other” (OR = 1.05, 95%CI = 0.763–1.456) populations than Black (OR = 0.38, 95%CI = 0.273–0.522) and Asian (OR = 0.40, 95%CI = 0.214–0.756) populations. Regular e-cigarette use was highest among those with high school degree (OR = 1.39, 95%CI = 1.101–1.756) or associate degree (OR = 1.37, 95%CI = 1.077–1.745) than those with a college degree or higher (OR = 0.81, 95%CI = 0.624–1.117) or less than 12 years of education (reference variable). Regular e-cigarette use was highest among those with a family income of $75,000 or more (OR = 1.09, 95%CI = 0.860–1.373) (not statistically significant).
McCabe et al., 2020	2015–2016 MTF survey	National	38,926 students (8th, 10th, 12th grades)	Cross-sectional	Use prevalence	Higher prevalence of ENDS use in schools with a higher percentage of White students (14.4% for 10th grade and 17.1% for 12th grade). Students who attended schools with the highest prevalence of past-month ENDS use had higher odds of past-month ENDS use (high prevalence of ENDS use, AOR = 6.82; 95% CI = 5.68, 7.96, *p* < 0.001: medium prevalence of ENDS use, AOR = 3.03; 95% CI = 2.60, 3.45, *p* < 0.001) than students who attended schools with the lowest prevalence of past-month ENDS use.
Moran et al., 2017	2016–2018 PATH W2	National	12,307 youth aged 12–17	Longitudinal	Advertisement	NHB youth reported using ENDS because the advertising appealed to them at over 2.5 and 3 times the rates of their Hispanic and NHW counterparts, respectively. Youth of lower SES less than high school: 52.3%, 95% CI = 40.21–64.13, high school graduate: 40.85%, 95% CI = 30.29–52.32, *p* = 0.0061) compared to youth of higher SES (some college education: 33.78%, 95%CI = 25.83–42.75, college degree or higher: 25.55%, 95%CI = 18.02–34.88, *p* = 0.0061), were more likely to report using ENDS products because people in the media or other public figures used them.
Quickstat 2019	2014 and 2018 NHIS	National	Sample size not provided; adults aged 18–24, 25–34, 35–44, 45–64, and ≥65	Cross-sectional	Use prevalence	Prevalence of use increased from 2014 to 2018 for all races (13.0–15.7%). Non-Hispanic white adults had the highest prevalence rate in both years (2018—19.1%).
Roberts et al., 2020	Midwestern university survey (2016 and 2018)	Local	529 students in 2016 and 611 students in 2018 aged ≥ 18	Prospective cohort	Use prevalence	Ever use of JUUL was associated with higher SES and being White. Likewise, past 30-day use of JUUL was higher among high-SES and White groups, although the effect was not always significant.
Simon et al., 2018	School survey	Local	7045 students (surveyed from eight high schools in Connecticut)	Cross-sectional	Advertisement	Indirect effect of SES and frequency (β = 0.01, SE = 0.00, 95%CI (0.001, 0.010), *p* = 0.02; B =.01, SE = 0.01, 95%CI (0.003, 0.022), *p* = 0.01) of ENDS use suggest youth of higher SES have greater recent advertising exposure, which is associated with greater frequency of ENDS use.
Spears et al., 2019	2016–2017 TPRPS	National	11,688 adults aged ≥ 18	Cross-sectional	Susceptibility	Among non-cigarette combustible tobacco users, young adults aged 18 to 29 years (OR = 6.5 95% CI = 4.3,8.8), those living below poverty (OR = 4.1 95% CI = 1.5, 6.8), those less educated (OR = 3.2 95% CI = 1.9, 4.6), those without health insurance (OR = 8.1 95%CI = 3.1, 13.1), those who identified as a sexual minority (OR = 3.5 95% CI = 0.9, 6.1), and NHB (OR = 2.7 95% CI = 1.0, 4.3) and Hispanic (OR = 3.3 95% CI = 1.2, 5.4) were more likely to use ENDS. Among cigarette users, those living at or above the FPL with higher education were more likely to use ENDS (OR (at or above FPL) = 60.7, 95%CI = 57.9–65.3) (OR (higher education) = 61.7, 95%CI = 58.4–64.9).
Springer et al., 2018	CMB	Local	5278 6th graders from 23 central Texas public middle schools	Cross-sectional	Use prevalence; susceptibility	Hispanic students reported significantly higher ENDS susceptibility (38.7% vs. 29.7%, *p* < 0.0001) and ever use (3% vs. 1.5%, *p* = 0.003) compared to White students. Students in the lowest SES schools were two times as likely to report ENDS susceptibility compared with students in the highest SES schools (adjusted odds ratio (AOR) = 2.01, 95% confidence interval (CI): 1.49–2.71).
Stallings–Smith and Ballantyne 2019	2015–2016 NHANES	National	5989 adults aged ≥ 18	Cross-sectional	Use prevalence	For non-smokers of conventional cigarettes, odds of ENDS use were higher among Hispanics compared with NHW, and non-working participants compared with those who were working. Odds of ENDS use were higher among those with less than high school education (OR = 1.47; 95%CI = 1.08–2.00) and incomes below the poverty level (OR = 1.31; 95%CI = 1.01–1.69).
Vallone et al., 2020	2018 TLC W7 and W8	National	14,379 (W7) and 12,114 (W8) participants aged 15–34	Longitudinal	Use prevalence	Use was highest among participants who were Hispanic (14.6% ever users and 6.8% current users) or white (14.4% ever users and 6.6% current users); identified as lesbian, gay, or bisexual (18.1% ever users and 8.9% current users); and lived in the northeast (17.1% ever users and 7.8% current users).
Venugopal et al., 2020	2018 Census Tract	National	10,989 school districts and 7479 vape shops	Spatial and statistical analyses	Access	Vape shops were further away from schools in districts with higher proportions of the population in poverty, but more densely distributed and in closer proximity to schools in districts with higher proportions of Asian and African American populations.
Vu et al., 2019	2017 A-TRAC	National	3000 participants (1549 ENDS users and 1451 never-ENDS users) aged 13–18	Cross-sectional	Susceptibility	Odds of perceiving harm from nicotine were 34% lower in NHB versus NHW, 33% lower in urban versus suburban residents, 40% higher in LGBTQ versus straight-identifying individuals, and 28% lower in low-income versus high-income families. Lower parental education level also was associated with children’s lower health risk perception of ENDS product contents.
Williams et al., 2020	2017 Nevada YRBS	State	5464 middle school students (6th through 8th grade)	Cross-sectional	Susceptibility	Higher odds of early initiation among Hispanic students versus NHW students (AOR = 1.89; 95%CI = 1.27–2.83), students residing in a rural county versus an urban county (AOR = 1.48; 95%CI = 1.02–2.14), and students living with a parent or another adult serving on active duty in the military (AOR = 1.72; 95% CI = 1.05–2.82). A graded relationship between the number of adverse childhood experiences (ACEs) and early initiation of ENDS products was also observed: 1 ACE (AOR = 1.60; 95%CI = 0.99–2.59), 2 ACEs (AOR = 2.29; 95%CI = 1.33–3.93), and 3–6 ACEs (AOR = 3.43, 95%CI = 2.20–5.36).
Wheeler et al., 2020	2012–2018 Virginia Census Tract	State	1820 census tracts, 5600 tobacco retail outlets, and 167 vape shops	Cross-sectional	Access	E-cigarette access was higher in neighborhoods with a higher percent Hispanic population, low household income, higher percent renter occupied housing, lower gross rent cost, and higher percent vacant housing.
Xiao et al., 2019	2016–2018 PATH W2	National	415 youth aged 12–17	Longitudinal	Advertisement; access	Participants with household incomes of less than $10,000 a year (16.27%), $10,000–$24,999 (26.42%), and $25,000–$49,999 (15.03%) reported using ENDS because the advertising appealed to them, versus 4.34% of those with household incomes of $100,000 or more. Non-Black and non-Hispanic ethnicities most commonly report use due to appealing flavors and have a higher likelihood of reporting ENDS use because of affordability compared with NHW. Other non-Hispanics reported e-cigarettes were affordable (AOR = 2.684, 95%CI = 1.044–6.899). Non-Hispanic “other” (89.30%), NHW (78.79%), and Hispanic (76.59%) youth reported higher ENDS use than NHB youth (53.62%) because the flavors appealed to them.
Yu and Lippert 2017	2014 NYTS	National	19,092 middle and high school students (public and private schools)	Cross-sectional	Use prevalence	Compared to NHW students, NHB and Asian students had lower odds of ENDS use (OR = 0.72, 95%CI = 0.63–0.83) (OR = 0.64, 95%CI = 0.50–0.81). In contrast, Hispanic and students of other races had higher odds of ENDS use (OR = 1.30, 95%CI = 1.1 –1.45) (OR = 1.17, 95%CI = 1.01–1.37). In schools where prevalence of ENDS use was high, the risk of individual ENDS use was higher among NHW than NHB.

**Notes:** AA–African American, ACS–American Community Survey, AI–American Indian, A-TRAC–American Heart Association Tobacco Regulation and Addiction Center, CA BOE–California Board of Equalization, CDTFA–California Department of Tax and Fee Administration, CHS–Southern California Children’s Health Study, CMB–Catch my breath, H&H–Happiness & Health Project, H/L–Hispanic/Latino, HINTS–Health Information National Trends Survey, KA–Korean American, LACHS–Los Angeles county health survey, MTF–Monitoring the future, NHANES–National Health and Nutrition Examination Survey, NHIS–National Health Interview Survey, NHW–Non-Hispanic White, NJ YTS–New Jersey Youth Tobacco Survey, PATH W2–Population Assessment of Tobacco and Health Wave 2, TEA–Texas Education Agency, TLC W7 and W8–Truth Longitudinal Cohort Wave 7 and Wave 8, TPRPS–Tobacco Products and Risk Perceptions Survey, YASS–Yale Adolescent Survey Study, YRBS–Youth Risk Behavior Survey, NYTS–National Youth Tobacco Survey. YRBSS–Youth Risk Behavior Surveillance System.

## Supplementary Materials

The following supporting information can be downloaded at: https://www.mdpi.com/article/10.3390/ijerph192013585/s1, Supplementary File S1: ENDS literature search terms; Supplementary File S2: Full-text extraction table.
