# Supplementary material for: Review of Use Prevalence, Susceptibility, Advertisement Exposure, and Access to Electronic Nicotine Delivery Systems among Minorities and Low-Income Populations in the United States
Source: Int J Environ Res Public Health. 2022 Oct 20;19(20):13585. doi: 10.3390/ijerph192013585 (PMC9603140; doi:10.3390/ijerph192013585)
Supplement: Supplementary file 1 [file ijerph-19-13585-s001.zip › Supplementary File1.pdf]

ENDS Literature Search Terms: 1/2020

PubMed

"Electronic nicotine devices"[TIAB] OR "Electronic nicotine device"[TIAB] OR "Electronic nicotine delivery systems"[TIAB] OR "Electronic nicotine delivery system"[TIAB] OR "electronic nicotine delivery device"[TIAB] OR "electronic nicotine delivery devices"[TIAB] OR "electronic cigarettes"[TIAB] OR "electronic cigarette"[TIAB] OR "e-cigarettes"[TIAB] OR "e-cigarette"[TIAB] OR e-cig[TIAB] OR e-cigs[TIAB] OR "ecig"[TIAB] OR "ecigs"[TIAB] OR "electric cigarettes"[TIAB] OR "electric cigarette"[TIAB] OR "vape cigarette"[TIAB] OR "vape cigarettes"[TIAB] OR e-liquid[TIAB] OR e-liquids[TIAB] OR "E liquid"[TIAB] OR "E liquids"[TIAB] OR eliquid*[TIAB] OR "vape juice"[TIAB] OR "nicotine liquid"[TIAB] OR "nicotine liquids"[TIAB] OR "liquid nicotine"[TIAB] OR "electronic hookah"[TIAB] OR "electronic hookahs"[TIAB] OR "hookah pen"[TIAB] OR "hookah pens"[TIAB] OR "e-hookah"[TIAB] OR "e-hookahs"[TIAB] OR "shisha pen"[TIAB] OR "shisha pens"[TIAB] OR "e-shisha"[TIAB] OR "e-shishas"[TIAB] OR "electronic cigar"[TIAB] OR "electronic cigars"[TIAB] OR "electric cigar"[TIAB] OR "electric cigars"[TIAB] OR (mods[TIAB] AND (vape[TIAB] OR vaping[TIAB] OR vapes[TIAB] OR vaper[TIAB] OR vapers[TIAB] OR vaped[TIAB])) OR e-juice[TIAB] OR e-juices[TIAB] OR (tank*[TIAB] AND (atomizer*[TIAB] OR vape[TIAB] OR vaping[TIAB] OR vapes[TIAB] OR vaper[TIAB] OR vapers[TIAB] OR vaped[TIAB])) OR vape[TIAB] OR vaping[TIAB] OR vapes[TIAB] OR vaper[TIAB] OR vapers[TIAB] OR vaped[TIAB] OR e-pipe[TIAB] OR "electronic pipe"[TIAB] OR "electronic pipes"[TIAB] OR "electric pipe"[TIAB] OR "electric pipes"[TIAB] OR "advanced personal vaporizer"[TIAB] OR "advanced personal vaporizers"[TIAB] OR "personal vaporizer"[TIAB] OR "personal vaporizers"[TIAB] OR vaporizer*[TIAB] OR "alternative cigarettes"[TIAB] OR "electronic nicotine inhalers"[TIAB] OR "electronic nicotine inhaler"[TIAB] OR "E smoke"[TIAB] OR "E smokes"[TIAB] OR "mechanical mod"[TIAB] OR "mechanical mods"[TIAB] OR "smokeless cigarettes"[TIAB] OR "vaporized nicotine"[TIAB] OR "vaporised nicotine"[TIAB] OR "aerosolize nicotine"[TIAB] OR "aerosolized nicotine"[TIAB] OR cig-a-like*[TIAB] OR cigalike*[TIAB] OR cartomizer*[TIAB] OR "electronic vaping device"[TIAB] OR juul*[TIAB] OR "nicotine salt"[TIAB] OR "nicotine salts"[TIAB] OR "pod mods"[TIAB] OR "pod system"[TIAB] OR "vape pods"[TIAB] OR "pod mod"[TIAB] OR "pod systems"[TIAB] OR "vape pod"[TIAB] OR "squonk mod"[TIAB] OR "bottom feeder mod"[TIAB] OR vaporette*[TIAB] OR "electronic vapour product"[TIAB] OR "electronic vapor cigarette"[TIAB] OR "electronic vaping devices"[TIAB] OR "vapour devices"[TIAB] OR "vapor devices"[TIAB] OR "electronic vapour products"[TIAB] OR "electronic vapor products"[TIAB] OR "electronic vapor product"[TIAB] OR "electronic vapor cigarettes"[TIAB] OR "alternative tobacco products"[TIAB] OR "emerging tobacco products"[TIAB] OR "Alternative Nicotine Delivery Systems"[TIAB] OR "Alternative Nicotine Delivery System" OR "Emerging nicotine delivery products"[TIAB] OR "Emerging nicotine delivery product"[TIAB] OR "Electronic Nicotine Delivery Systems"[Mesh] OR "Vaping"[Mesh] OR "vaporized nicotine products"[TIAB] OR "nicotine vaping products"[TIAB] OR "vaporized nicotine product"[TIAB] OR "nicotine vaping product"[TIAB] OR "vaporized nicotine"[TIAB] OR "vaporised nicotine"[TIAB]

Embase

"Electronic nicotine device*" OR "Electronic nicotine delivery system*" OR "electronic nicotine delivery device*" OR "electronic cigarette*" OR "e cig*" OR ecig* OR "electric cigarette*" OR "vape cigarette*" OR "e liquid*" OR eliquid* OR "vape juice*" OR "nicotine liquid*" OR "liquid nicotine" OR "electronic hookah*" OR "e hookah*" OR "hookah pen*" OR "shisha

pen*” OR “e shisha*” OR “electronic cigar” OR “electric cigar” OR mods NEAR/5 vap* OR “e juice*” OR tanks NEAR/5 atomizer* OR tanks NEAR/5 vap* OR vape OR vapes OR vaper OR vapers OR vaped OR vaping OR “e pipe*” OR “electronic pipe” OR “electric pipe” OR “advanced personal vaporizer” OR “personal vaporizer” OR vaporizer OR “alternative cigarette” OR “electronic nicotine inhaler” OR “E smoke” OR “mechanical mod” OR “smokeless cigarette” OR "vapo?rized nicotine" OR "aerosoli?e nicotine" OR “cig a like*” OR cigalike OR cartomizer* OR ”electronic vaping device*” OR “electronic vapour product*” OR “electronic vapor cigarette*” OR “vapor device*” OR “vapour device*” OR “electronic vapor product*” OR juul*:ti,ab OR “nicotine salt*” OR “pod mod” OR “pod mods” OR “pod system*” OR “vape pod*” OR “squonk mod*” OR “bottom feeder mod*” OR vaporette* OR “alternative tobacco product*” OR “emerging tobacco product*” OR “Alternative Nicotine Delivery System*" OR “Emerging nicotine delivery product?” OR “Electronic Cigarette"/exp OR Vaping/exp OR “vaporized nicotine products”:ti,ab OR “nicotine vaping products”:ti,ab OR “vaporized nicotine product”:ti,ab OR “nicotine vaping product”:ti,ab OR “vapori?ed nicotine”

WOS*
*Note: copying and pasting tends to erase some of the quotation marks so please double check the search strategy after pasting into Web of Science before running or creating email alert.

“Electronic nicotine device*" OR "Electronic nicotine delivery system*" OR “electronic nicotine delivery device*” OR "electronic cigarette*" OR "e-cigarette*" OR e-cig* OR ecig* OR “electric cigarette*” OR “vape cigarette*” OR e-liquid* OR “E liquid*” OR eliquid* OR “vape juice” OR “nicotine liquid*” OR “liquid nicotine” OR “electronic hookah*” OR “e-hookah*” OR “hookah pen*” OR “shisha pen*” OR “e-shisha*” OR “electronic cigar*” OR “electric cigar*” OR mods NEAR/5 vap* OR e-juice* OR tanks NEAR/5 vap* OR tanks NEAR/5 atomizer* OR vape OR vapes OR vaper OR vapers OR vaped OR vaping OR e-pipe OR “electronic pipe*” OR “electric pipe*” OR “advanced personal vaporizer*” OR “personal vaporizer*” OR vaporizer* OR “alternative cigarette*” OR “electronic nicotine inhaler*” OR “E smoke*” OR “mechanical mod?” OR “smokeless cigarette*” OR "vapo*rized nicotine" OR "aerosoli*e nicotine" OR cig-a-like* OR cigalike OR cartomizer* OR ”electronic vaping device*” OR “electronic vapour product*” OR “electronic vapor cigarette*” OR “vapor device*” OR “vapour device*” OR “electronic vapor product*” OR juul* OR “nicotine salt*” OR “pod mod” OR “pod mods” OR “pod system*” OR “vape pod*” OR “squonk mod*” OR “bottom feeder mod*” OR vaporette* OR “alternative tobacco product*” OR “emerging tobacco product*” OR “alternative nicotine delivery system*" OR “emerging nicotine delivery product*” OR “vaporized nicotine product*” OR “nicotine vaping product*” OR “vapori?ed nicotine”

Derwents
“Electronic nicotine device*" OR "Electronic nicotine delivery system*" OR “electronic nicotine delivery device*” OR "electronic cigarette*" OR "e-cigarette*" OR e-cig* OR ecig* OR “electric cigarette*” OR “vape cigarette*” OR e-liquid* OR “E liquid*” OR eliquid* OR “vape juice” OR “nicotine liquid*” OR “liquid nicotine” OR “electronic hookah*” OR “e-hookah*” OR “hookah pen*” OR “shisha pen*” OR “e-shisha*” OR “electronic cigar*” OR “electric cigar*” OR mods NEAR/5 vap* OR e-juice* OR tanks NEAR/5 vap* OR tanks NEAR/5 atomizer* OR vape OR vapes OR vaper OR vapers OR vaped OR vaping OR e-pipe OR “electronic pipe*” OR “electric pipe*” OR “advanced personal vaporizer*” OR “personal vaporizer*” OR “alternative cigarette*” OR “electronic nicotine inhaler*” OR “E smoke*” OR “mechanical mod?” OR “smokeless cigarette*” OR "vapo*rized nicotine" OR "aerosoli*e nicotine" OR cig-a-like* OR cigalike OR cartomizer* OR ”electronic vaping device*” OR

ENDS Literature Search Terms: 1/2020

“electronic vapour product*” OR “electronic vapor cigarette*” OR “vapor device*” OR “vapour device*” OR “electronic vapor product*” OR juul* OR “nicotine salt*” OR “pod mod” OR “pod mods” OR “pod system*” OR “vape pod*” OR “squonk mod*” OR “bottom feeder mod*” OR vaporette* OR “alternative tobacco product*” OR “emerging tobacco product*” OR “alternative nicotine delivery system*" OR “emerging nicotine delivery product*” OR “vaporized nicotine product*” OR “nicotine vaping product*” OR “vapori?ed nicotine”
AND
tobacco OR nicotine OR flavor* OR flavour*

EBSCOHost (Suggested databases: Academic Search Complete, PsycINFO, Science & Technology Collection)

“Electronic nicotine device?" OR "Electronic nicotine delivery system?" OR “electronic nicotine delivery device?” OR "electronic cigarette?" OR "e-cigarette?" OR e-cig? OR ecig? OR “electric cigarette?” OR “vape cigarette?” OR e-liquid? OR “E liquid?” OR eliquid* OR “vape juice” OR “nicotine liquid?” OR “liquid nicotine” OR “electronic hookah?” OR “e-hookah?” OR “hookah pen?” OR “shisha pen?” OR “e-shisha?” OR “electronic cigar?” OR “electric cigar?” OR mods N5 vap* OR e-juice? OR tanks N5 vap* OR vape OR vapes OR vaper OR vapers OR vaped OR vaping OR e-pipe OR “electronic pipe?” OR “electric pipe?” OR “advanced personal vaporizer?” OR “personal vaporizer?” OR vaporizer? OR “alternative cigarette?” OR “electronic nicotine inhaler?” OR “E smoke?” OR “mechanical mod?” OR “smokeless cigarette?” OR "vapo?rized nicotine" OR "aerosoli?e nicotine" OR cig-a-like* OR cigalike OR cartomizer* OR ”electronic vaping device?” OR “electronic vapour product?” OR “electronic vapor cigarette?” OR “vapor device?” OR “vapour device?” OR “electronic vapor product?” OR juul* OR “nicotine salt*” OR “pod mod?” OR “pod system*” OR “vape pod*” OR “squonk mod*” OR “bottom feeder mod*” OR vaporette* OR “alternative tobacco product?” OR “emerging tobacco product?” OR “Alternative Nicotine Delivery System?" OR “Emerging nicotine delivery product?” OR “vaporized nicotine product*” OR “nicotine vaping product*” OR “vapori?ed nicotine”

SciFinder (Create Keep Me Posted for each)
Electronic nicotine delivery system
e-cigarette
Electronic cigarette
Electronic hookah
Electronic cigar
e-liquid (refined with tobacco OR nicotine OR flavor OR flavor)
vaping
